# Patient safety culture in a district hospital in South Africa: An issue of quality

**DOI:** 10.4102/curationis.v38i1.1518

**Published:** 2015-11-05

**Authors:** Lorraine M. Mayeng, Jacqueline E. Wolvaardt

**Affiliations:** 1 National District Hospital, Bloemfontein, South Africa; 2 School of Health Systems and Public Health, University of Pretoria, South Africa

## Abstract

**Background:**

The *Nursing Act* 33 of 2005 holds nurse practitioners responsible for all acts and omissions in the delivery of quality patient care. But quality patient care is influenced by a number of factors beyond the control of nurse practitioners. Patient safety culture is one such factor and is seldom explored in hospitals in developing countries. This article describes the patient safety culture of a district hospital in South Africa.

**Objectives:**

The study identified and analysed the factors that influence the patient safety culture by using the Manchester Patient Safety Framework at the National District Hospital, Bloemfontein, Free State Province.

**Method:**

A descriptive cross-sectional study was conducted and included the total population of permanent staff; community service health professionals; temporarily employed health professionals and volunteers. The standard Manchester Patient Safety Framework questionnaire was distributed with a response rate of 61%.

**Results:**

Less than half of the respondents (42.4%; *n* = 61) graded their units as acceptable. Several quality dimensions were statistically significant for the employment profile: overall commitment to quality (*p* = 0.001); investigating patient incidents (*p* = 0.031); organisational learning following incidents (*p* < 0.001); communication around safety issues (*p* = 0.001); and team working around safety issues (*p* = 0.005). These same quality dimensions were also statistically significant for the professional profiles. Medical doctors had negative perceptions of all the safety dimensions.

**Conclusion:**

The research measured and described patient safety culture (PSC) amongst the staff at the National District Hospital (NDH). This research has identified the perceived inadequacies with PSC and gives nurse managers a clear mandate to implement change to ensure a PSC that fosters quality patient care.

## Introduction

Patient safety is at the forefront of service delivery in South Africa (SA), as the health system is struggling to cope with the collision of four excessive health burdens, namely communicable diseases (especially HIV/AIDS); non-communicable diseases; maternal, neonatal and child deaths; and deaths from injury and violence (Coovadia et  al. 2009:817–843). At the same time, SA experiences acute shortages of health professionals in the publicly funded sector. This combination of increasing numbers of patients and a shortage of professionals is a real concern for nurse managers, as nurse practitioners are responsible for all acts and omissions in the delivery of quality patient care (Eygelaar & Stellenberg 2012:1) and patients have little guarantee that they are receiving safe health care.

Many healthcare quality problems have been identified in both the private and public sectors in SA. The most notable are: under- and over-use of services; avoidable errors; lack of resources; inadequate diagnosis and treatment; inefficient use of resources; and drug shortages and poor delivery systems (National Department of Health 2007:3). In July 2009 the SA Ministry of Health released a programme of action which comprised ten priority actions to address the service delivery challenges. One of the key priorities of this plan is the improvement in the quality of healthcare services (National Department of Health 2010:6).

The most common problems experienced by healthcare users in public institutions include: lack of cleanliness; poor safety and security; long waiting times; poor staff attitude; and poor infection control measures and the non-availability of drugs (National Department of Health 2011a:4). In summary all these issues indicate possible poor quality of care.

Quality care can be defined in the light of the provider’s technical standards but also patients’ expectations. Quality is a comprehensive and multifaceted concept, and dimensions of quality include technical competence; access to service; effectiveness; interpersonal relations; efficiency; continuity of care; safety and amenities (Brown et  al. 1993:8–10). Nurse managers need to be able to meet these provider- and patient expectations within policy and fiscal constraints.

Patient safety is a dimension of quality assurance. ’Patient safety practices‘ refers to those processes or structures which, when applied, reduce the probability of adverse events resulting from exposure to the healthcare system across a range of diseases and procedures (World Health Organization 2008:1). Furthermore, patient safety includes initiatives to identify, report, analyse and prevent any unintended or unexpected incidents that could harm healthcare users (National Department of Health 2011b:22).

Patient safety is a serious global public health issue and patient safety is a top priority for action by the Agency for Healthcare Research and Quality in the United States of America (Kronick 2014:196). Unsafe patient care is associated with significant morbidity and mortality rates throughout the world, and much of it might be amenable to timely intervention (World Health Organization, World Alliance for Patient Safety 2008:3). In developing countries the probability of a patient being harmed in hospitals is high, with the risk of healthcare-associated infection as much as 20 times higher than in developed countries (World Health Organization n.d., fact 3). Wilson et  al. (2012:1–14) conducted a study in eight developing countries, including SA. The aim of the study was to assess both the frequency and the nature of adverse events experienced by patients. The results indicated that of the 15 548 records reviewed 8.2% showed at least one adverse event, with a range of 2.5% to 18.4% per country. 83% of those adverse events were preventable and 30% were associated with the death of the patients (Wilson et  al. 2012:4.). Key to ensuring patient safety is ensuring a patient safety culture (PSC) in the organisation.

### Problem statement

#### Literature review

The ultimate goal of a health system is to improve peoples’ health by providing comprehensive, integrated, equitable, quality and responsive health services (World Health Organization 2010:14). Quality health care can be provided in health care facilities only if the health system of the country achieves its strategic goals of improving, promoting, restoring and maintaining health.

In his ground-breaking work in the 1980s, Donabedian created a framework for measuring quality in health care. Key to his work are the concepts of ’quality assessment‘ (measurement of quality care) and ’quality assurance‘ (improving the quality of care). He suggests three approaches to assessing quality of care namely structure, process and outcome (Donabedian 1980:1).

Structure refers to the material resources such as facilities and equipment, human resources such as the number, variety and qualification of personnel and organisational characteristics, including the kinds of supervision and performance review as well as methods of paying for care.

Process, on the other hand, includes the activities that constitute health care, such as diagnosis and treatment, usually carried out by professional personnel but also by patients and family.

Finally, outcome refers to the changes in individuals attributable to the care they received such as changes in health status, changes in behaviour of patients and family members and changes in knowledge acquired by them as well as the satisfaction of patients and family members.

Patient safety is a dimension of quality assurance, and needs to be maintained in a health system. Patient safety practises refer to those processes or structures which when applied reduce the probability of adverse events resulting from exposure to the healthcare system across a range of diseases and procedures (World Health Organization 2008:1) Patient safety includes initiatives to identify, report, analyse and prevent any unintended or unexpected incidents that could harm healthcare users (National Department of Health 2011b:22). Organisational culture plays an important role in patient safety practices.

#### Patient safety culture

The organisational culture inherent in every healthcare organisation often has more of an impact on patient safety than any problems that are related to process (Spath 2001:85. In order to create a PSC, health professionals must value following those practices that promote patient safety.

The Institute of Medicine in the United States of America suggests that the biggest challenge to moving towards a safer healthcare system is changing the PSC from one in which people are blamed for errors to one in which errors are treated as opportunities to improve the system and avoid harm (Institute of Medicine 2001:2). Leadership is the most important element in a successful patient safety programme and leadership cannot be delegated (Botwinick, Bisognano & Haraden 2005:1–37). The NDH in SA has experienced some adverse events that have resulted in the perception that the hospital is unsafe. A first step to addressing these quality concerns was to assess the current PSC using the Manchester Patient Framework.

#### The Manchester Patient Safety Framework 

The MaPSaF is a tool that was developed by Parker *et al.* in 2006 and was developed through extensive healthcare literature reviews and consultations with healthcare professionals (Law et  al. 2010:110). The MaPSaF makes the concept of a ‘safety culture’ more accessible to healthcare teams and organisations. Originally designed for use by general practices and primary care setting, the MaPSaF has now been adapted for use in other healthcare settings. The tool describes nine quality dimensions (Table 1) and was tested and validated in acute care settings in Canada and the United Kingdom (Law et  al. 2010:111).

**TABLE 1:** The nine quality dimensions of the Manchester Patient Safety Framework.

Number	Dimensions	Description
1	Overall commitment to quality	How much is invested in developing the quality agenda?
		What is seen as the main purpose of policies and procedures?
		What attempts are made to look beyond the organisation for collaboration and innovation?
2	Priority given to patient safety	How seriously is the issue of patient safety taken within the organisation?
		Where does responsibility lie for patient safety issues?
	Perceptions of the causes of patient safety incidents and their identification	What sort of reporting system is there?
		How are reports of incidents received?
		How are incidents viewed as an opportunity to blame or improve?
4	Investigating patient safety incidents	Who investigates incidents and how are they investigated?
		What is the aim of the organisation? Does the organisation learn from the event
5	Organisational learning following a patient safety incident	What happens after an incident?
		What mechanisms are in place to learn from the incident?
		How are changes introduced and evaluated?
6	Communication about safety issues	What communications systems are in place?
		What are their features?
		What is the quality of record-keeping to communicate about safety like?
7	Personnel management and safety issues	How are safety issues managed in the workplace?
		How are staff problems managed? What are the recruitment and section procedures like?
8	Staff education and training about safety issuessss	How, why and when are education and training programmes about patient safety developed?
		What do staff think of them?
9	Team working around safety issues	How and why are teams developed?
		How are teams managed?
		How much team working is there around patient safety issues?

The MaPSaF outlined in Table 1 was developed to help make the concept of safety culture more accessible to healthcare teams and to assist organisations to understand their level of development with respect to the value that they place on patient safety (National Health Service 2006:1). Since the completion of this study, these quality dimensions were refined resulting in a list with 10 dimensions.

The tool helps healthcare teams and organisations reflect on their progress in developing a mature safety culture (National Health Service 2006:1). The MaPSaF was chosen to assess the PSC at the National District Hospital (NDH) as an opportunity to foreground quality concerns within the hospital. The MaPSaF is not designed to be used for performance management or assessment purposes, or to assign blame when the results show that an organisation’s safety culture is not yet satisfactorily mature. This characteristic of not assigning blame was important in the methodology.

## Research method and design

### Design

The study design was a quantitative descriptive cross-sectional study. The choice of study design was based on the need to determine a baseline of the patient safety climate at the hospital.

### Materials

The standard MaPSaF questionnaire contains mostly closed-ended questions and was expanded to include information on demographic variables and background information. The order of the questions within the questionnaire was randomised. The questionnaire was translated into South Sotho by a linguist and back translated to English to ensure that the validity of the questions remained unchanged. South Sotho was included, as it is commonly spoken in the area. The questionnaire was available in both English and South Sotho.

### Data collection method

Data collection was undertaken by the principal investigator (as the head of nursing) and two external field workers amongst the personnel of the NDH. The field workers were responsible for the distribution and collection of questionnaires and assisted those participants who needed help with completing the questionnaire.

As PSC is an organisational phenomenon, both clinical and non-clinical departments were included in the study. The clinical staff consisted of: medical doctors; nurses; and clinical support (dieticians, physiotherapists, occupational therapists, pharmacists and radiographers). Participants in the study included all permanent staff (clinical and non-clinical); health professionals busy with their compulsory community service; temporarily employed health professionals (e.g. session and agency staff) and volunteers (e.g. lay counsellors and home-based carers). All units in the hospital were briefed on the project. Staff members were also briefed in the routine meetings such as the management, finance, nurses and quality assurance meetings leading up to the start of the project. Only staff members who had work experience at the NDH for at least six months were invited to participate. Both day and night staff members were included. The study was conducted from November 2010 to July 2011. At the time of the data collection there were 381 members of staff, and 200 questionnaires were distributed to those who met the inclusion criteria. Staff members who had been working at the NDH for less than six months or who were on leave (maternity, annual, sick or training), were not included.

## Data analysis

The collected data were captured using Epidata software and analysis was done with the Stata package. Responses were indicated on a five-point Likert scale, with the most positive responses of ‘strongly agree’ allocated the highest point of 5 and the most negative responses of ‘strongly disagree’ allocated the lowest point of 1. During analysis new variables were created: ‘strongly agree’ and ‘agree’ were combined and named ‘positive’, ‘neither’ became ‘neutral’ and ‘strongly disagree’ and ‘disagree’ became ‘negative’. Univariate regression was done to determine the responses of the different categories of health professionals and the responses of the different employment categories of the participants. *P*-values were determined with a 95% level of significance. All negatively-worded questions were reverse coded during the data analysis process.

## Context of the study

The NDH was established in 1998 as an initiative of the Free State Department of Health in adopting a primary health care approach as a vehicle for providing District Health Care Services.

This level 1 hospital is situated in Bloemfontein in the Motheo district; its catchment area includes Naledi sub-district (Dewetsdorp, Wepener, Van Stadenrus) and other soft border areas like Boshoff, Brantfort Deasville and Soutpan which fall under the Letsweleputsa district. Patients from these areas make use of the NDH although strictly speaking they are outside the catchment area. The hospital supports two community health centres in Bloemfontein and is a referral hospital for 44 clinics. The hospital refers to the Pelonomi Regional Hospital and Universitas Academic Hospital.

The catchment population of NDH is 736 158, of whom 80.3% are uninsured, placing a substantial burden on the hospital.

Services rendered include: 24-hour services; emergency; maternity; general; surgical; victim centre for rape victims; paediatric; radiology; step-down facilities and operating theatre services. The following are provided on an outpatient basis: dentistry; occupational therapy; physiotherapy; dietetics; social work; anti-retroviral services; psychology; and medical and surgery out-patients clinics.

The NDH is the only district hospital in the province with an academic platform for the University of the Free State. The NDH offers placement in training for registrars in Family Medicine, final year medical students and interns. The hospital is also a placement site for the University of the Free State Health Department and for the Free State Nursing College South sub-campus.

### Results

The overall response rate was 72% (*n*/*N* = 144/200). The demographic background of the respondents (Table 2) is typical of the South African health professional workforce: mostly female (75%; *n* = 108), black people (71.5%; *n* = 103), nurses (61.8%; *n* = 89) with post-secondary school qualifications (71.5%; *n* = 103).

**TABLE 2:** Demographic profile of participants.

Variable	Category	Number of participants	Percentage
Gender	Male	36	25.0
	Female	108	75.0
Education	Primary school	2	1.4
	Secondary school	39	27.1
	Tertiary	68	47.2
	Postgraduate	35	24.3
Ethnicity	Black people	103	71.5
	White people	31	21.5
	Mixed race people	8	5.6
	Indian people	1	0.7
	Other people	1	0.7
Work experience	Less than 1 year	46	31.9
	1–5 years	53	36.8
	6–10 years	45	31.3
Staff category	Medical	24	16.7
	Nursing	89	61.8
	Clinical supportss	12	8.3
	Admin & Support	19	13.2
Employment contract	Permanent	99	68.8
	Session	5	3.5
	Agency	9	6.3
	Community service	31	21.3
Contact with patients	Yes	130	90.3
	No	14	9.7

The majority of respondents held permanent appointments (68.8%; *n* = 99), and had at least one year’s experience (68.1%; *n* = 98), whilst a substantial proportion (31.3%; *n* = 45) had considerable work experience of more than six years.

## Health professional profile and the Manchester Patient Safety Framework dimensions

For the purpose of this study, analysis of the dimensions according to the health professional profile and employment status (permanent versus contract) was done. These two factors have a direct impact on clinical patients’ safety issues. From the principal investigator’s experience in the study setting, the majority of adverse events reported involved health professionals working directly with patients. The prevailing general perception in the organisation is that some of the adverse events resulted when contracted personnel (agency and sessional staff) were on duty.

Doctors were consistently negative about all nine patient safety dimensions, whilst nurses were lukewarm in their responses on eight of the dimensions (Table 3).

**TABLE 3:** Analysis of the nine quality dimensions and professional profile.

Dimension	Medical *N* = 24 *n* (%)	Nursing *N* = 89 *n* (%)	Clinical support *N* = 12 *n* (%)	*P*-value
Negative	16 (66.7)	29 (32.6)	7 (58.3)	0.031
Neutral	2 (8.3)	15 (16.9)	1 (8.3)	
Positive	6 (25.0)	49 (50.6)	4 (33.3)	
Negative	17 (70.8)	37 (41.6)	4 (33.3)	0.115
Neutral	1 (4.2)	7 (7.9)	1 (8.3)	
Positive	6 (25.0)	45 (50.6)	7 (58.3)	
Negative	17 (70.8)	44 (49.4)	5 (41.7)	0.296
Neutral	0 (0.0)	4 (4.5)	1 (8.3)	
Positive	7 (29.2)	41 (46.1)	6 (50.0)	
Negative	16 (66.7)	32 (36.0)	6 (50.0)	0.028
Neutral	4 (16.7)	14 (15.7)	0 (0.0)	
Positive	4 (16.7)	43 (48.3)	6 (50.0)	
Negative	17 (70.8)	21 (23.6)	6 (50.0)	0.000
Neutral	2 (8.3)	12 (13.5)	3 (25.0)	
Positive	5 (20.8)	56 (62.9)	3 (25.0)	
Negative	17 (70.8)	35 (39.3)	8 (66.7)	0.046
Neutral	2 (8.3)	10 (11.2)	1 (8.3)	
Positive	5 (20.8)	44 (49.4)	3 (25.0)	
Negative	15 (62.5)	44 (49.4)	4 (33.3)	0.325
Neutral	0 (0.0)	9 (9.0)	1 (8.3)	
Positive	9 (37.5)	39 (41.6)	7 (58.3)	
Negative	14 (58.3)	36 (40.5)	5 (41.7)	0.330
Neutral	5 (20.8)	13 (14.6)	2 (16.7)	
Positive	5 (20.8)	40 (44.9)	5 (41.7)	
Negative	16 (66.7)	27 (30.3)	6 (50.0)	0.019
Neutral	2 (8.3)	12 (13.5)	2 (16.7)	
Positive	6 (25.0)	50 (56.2)	4 (33.3)	

Only organisational learning following a patient safety incident was scored substantially positively by the nurses (62.9%; *n* = 56). Within their group, doctors scored the dimension on staff education and training about safety issues the least poorly (58.3%; *n* = 14). The clinical support team was the most negative about communication on safety issues (66.7%; *n* = 8), and within this group the most positive about the priority given to patient safety (58.3%; *n* = 7), as well as personnel management and safety issues (58.3%; *n* = 7).

There were five domains where the results were significant: overall commitment to quality dimension (*p* = 0.031); investigating patient safety incidents (*p* = 0.028); organisational learning following a patient safety incident (*p* < 0.001); communication about safety issues (*p* = 0.046); and team working around safety issues (*p* = 0.019).

## Employment profile and the Manchester Patient Safety Framework dimensions

The responses on the safety dimensions were analysed according to employment status in three groups: permanent, temporary (agency nurses and session doctors) and community service (Table 4).

**TABLE 4:** Analysis of the nine quality dimensions and employment status.

Dimension	Permanent *N* = 99 *n* (%)	Temporary *N* = 14 *n* (%)	Community service *N* = 31 *n* (%)	*P*-value
**Overall commitment to quality dimension**				
Negative	37 (37.4)	1 (7.1)	22 (71.0)	0.001
Neutral	14 (14.1)	2 (14.3)	3 (9.7)	
Positive	48 (48.5)	11 (78.6)	6 (19.4)	
**Priority given to patient safety**				
Negative	41 (41.4)	5 (35.7)	19 (61.3)	0.269
Neutral	8 (8.1)	1 (7.1)	3 (9.7)	
Positive	50 (50.5)	8 (57.1)	9 (29.0)	
**Perceptions of the causes of patient safety incidents and their identification**				
Negative	46 (46.5)	4 (28.6)	19 (61.3)	0.084
Neutral	8 (8.1)	0 (0.0)	0 (0.0)	
Positive	45 (45.5)	10 (74.4)	12 (46.7)	
**Investigating patient safety incidents**				
Negative	44 (44.4)	1 (7.1)	16 (51.6)	0.031
Neutral	11 (11.1)	3 (21.4)	6 (19.4)	
Positive	44 (44.4)	10 (71.4)	9 (29.0)	
**Organisational learning following a patient safety incident**				
Negative	29 (29.3)	1 (7.1)	19 (61.3)	0.000
Neutral	10 (10.1)	5 (35.7)	3 (9.7)	
Positive	60 (60.6)	8 (57.1)	9 (29.0)	
**Communication about safety issues**				
Negative	36 (36.4)	4 (28.6)	23 (74.2)	0.001
Neutral	10 (10.1)	1 (7.1)	4 (12.9)	
Positive	53 (53.5)	9 (64.3)	4 (12.9)	
**Personnel management and safety issues**				
Negative	46 (46.5)	6 (42.9)	17 (54.8)	0.633
Neutral	9 (9.1)	0 (0.0)	2 (6.5)	
Positive	44 (44.4)	8 (57.1)	12 (38.7)	
**Staff education and training about safety issues**				
Negative	44 (44.4)	4 (28.6)	17 (54.8)	0.108
Neutral	15 (15.2)	1 (7.1)	7 (22.6)	
Positive	40 (40.4)	9 (64.3)	7 (22.6)	
**Team working around safety issues**				
Negative	30 (30.3)	3 (21.4)	20 (64.5)	0.005
Neutral	11 (11.1)	3 (21.4)	3 (9.7)	
Positive	58 (55.6)	8 (57.1)	8 (25.8)	

The community service group gave negative responses across almost all the dimensions compared to the temporary group, who responded positively. Most of the responses given by the permanent group were average over the dimensions, except for organisational learning following a patient safety incident dimension that scored highly 60.6% (*p* < 0.001).

Overall, the results indicated that the community service staff had poor opinions on almost all nine dimensions, with the score on communication about safety issues scoring particularly poorly at 74.2% (*p* = 0.001).

The results of five dimensions were significant, namely: overall commitment to quality (*p* = 0.001); investigating patient safety issues (*p* = 0.031); organisational learning following a patient safety incident (*p* < 0.01); communication about safety issues (*p* = 0.01); and team work around safety issues (*p* = 0.005).

## Univariate regression results of the association between the nine Manchester Patient Safety Framework dimensions and the demographic variables

Owing to the small sizes the variables ’Mixed race people‘, ’Indian people‘ and ’other‘ were merged in the ethnic grouping. The perceptions of the white respondents were statistically significantly less favourable across all nine quality dimensions (*p*-value ranged between 0.011 and < 0.001). The other combined respondents also had a highly significant poor perception of quality compared to black people on dimensions of: perceptions of the causes of patient safety incidents (*p* < 0.001); communication about safety issues (*p* < 0.001); and personnel management and safety issues (*p* < 0.001).

The nurses’ positive perceptions were significant for perceptions of the causes of patient safety incidents (*p* < 0.003); investigating patient safety incidents (*p* < 0.001); and organisational learning following a patient safety incident (*p* < 0.001). The community service professionals had a significantly negative perception compared to the permanent staff on the dimensions: overall commitment to quality dimension (*p* < 0.001); organisational learning following a patient safety incident (*p* < 0.001); and communication about safety issues dimensions (*p* < 0.001).

## Overall view of patient safety in the hospital

The respondents were asked to grade their own unit on patient safety on a five-point Likert scale. The score allocation was allocated as: 1 = failing, 2 = poor, 3 = acceptable, 4 = very good and 5 = excellent. Less than half of the respondents (42.4%; *n* = 61) graded their units as acceptable. Few graded their units as very good (28.5%; *n* = 41) and even fewer as excellent (14.6%; *n* = 21). Fortunately the number who thought their units were poor (11.8%; *n* = 17) and failing (2.8%; *n* = 4) were in the minority.

## Ethical considerations

Ethical approval was granted from the ethics committee of the University of Pretoria (201/2010), and permission was provided by the Free State Provincial Department of Health.

## Potential benefits and hazards

A potential hazard for participants was identified as risk or fear of retaliation from hospital management for providing a poor opinion. A briefing session was therefore held to answer questions and to describe the anonymous and voluntary nature of participation. The questionnaire was anonymous and participants could not be identified. Only aggregated data are reported.

### Informed consent

The anonymous questionnaire was prefaced by an information letter to the participants. This letter emphasised voluntary participation, the right of refusal to participate and the right to withdraw once started. Furthermore the letter outlined the possible benefits and hazards for themselves and underlined the anonymous nature of their participation. Participants were invited to ask questions to clarify any uncertainties.

### Data protection

The hard copies of the questionnaire were securely stored in the principal investigator’s office until handed over to the University of Pretoria for further storage. The statistician only had access to the amalgamated electronic file of responses. Electronic files were password-protected.

## Trustworthiness

### Reliability and validity

The MaPSaF is a standardised tool and has been previously tested for reliability and validity in various settings (Law et  al. 2010:111).

## Discussion

### Outline of the results

The respondents felt that patient safety incidents were not investigated and that there was a lack of commitment to quality issues. This finding is a particular concern when viewed in the light of the need to identify, report, analyse and prevent any unintended or unexpected incidents that could harm healthcare users (National Department of Health 2011b:22). These perceptions can be supported by the low reporting of incidents within the current reporting system. The medical doctors – both permanent and community service – had negative perceptions about all the safety dimensions, with personnel management on safety issues scoring less poorly than the other dimensions. These perceptions could be attributed to the minimal participation of this group in quality improvement programmes and poor attendance of hospital staff meetings. These perceptions could also be a reflection of medical doctors’ expectations with regard to the quality dimensions of technical competence, safety and the amenities available at the NDH (Brown et  al. 1993:8–10).

The nurses’ positive perceptions regarding: the causes of patient safety incidents; investigating patient safety incidents; and organisational learning following a patient safety incident could be attributed to the fact that nurses are normally required to account for all the incidents that happen in the units and during this process they learn and work as a team. The negative perceptions of the community service professionals are possibly due to their lack of experience in clinical settings or peripheral engagement with issues that are not directly related to their patients’ care.

One positive feature was the positive response by the nurses on the organisational learning following an incident. This finding indicates that, although incidents are taking place as reported by the community and media, the nurses are learning from these incidents and the ’at risk behaviour‘ is identified (Clarke, Lerner & Marcella 2007:312).

There are clear differences in the perceptions of the different ethnic respondents about patient safety at the NDH. These differences can possibly be attributed to different expectations of what constitutes good quality care (Brown et  al. 1993:8–10) or possible prior experiences in academic hospitals, which are better resourced than district hospitals.

The more positive perception by the nurses over the spectrum of the quality dimensions could be as a result of greater intimacy with the system. The findings showed that systems are in place, but the personnel are not yet fully committed to attending to patient safety issues.

The NDH is registered with the Council of Health Service Accreditation of South Africa (COHSASA) that assists health institutions to meet and maintain quality standards (COHSASA 2014). Association with COHSASA has assisted in the development of quality assurance programmes and the development of standards. The findings on the PSC are a true reflection of the presence of quality assurance programmes that are not fully mature enough to create a safe patient environment.

### Practical implication

It is clear that PSC needs to be improved at the NDH and this improvement can be fostered by stronger nursing leadership in promoting a mature PSC. Nursing management should be trained in PSC assessment and involved in hospital walking rounds to communicate and build awareness of the dimensions of safety issues with all staff.

## Limitations of the study

As the study is a single institution study results cannot be generalised to other institutions.

## Recommendations

It is recommended that nurse managers participate in PSC workshops and are trained in the assessment and development of PSC within the NDH. Regular reviews of PSC should become a standard nursing management feature. The MaPSaF could further be used in workshops sessions to raise awareness of the current strengths and weakness so as to target improvement plans.

## Conclusion

This research successfully measured and described PSC amongst the various categories of staff at the NDH. This research has identified the perceived inadequacies with PSC and is a basis for a quality improvement project within the hospital.
